# Retraction Note: Applications of complex picture fuzzy soft power aggregation operators in multi-attribute decision making

**DOI:** 10.1038/s41598-025-14388-z

**Published:** 2025-08-04

**Authors:** Tahir Mahmood, Zeeshan Ali, Muhammad Aslam

**Affiliations:** 1 Department of Mathematics & Statistics, International Islamic University Islamabad, Islamabad, Pakistan; 2 Department of Mathematics, College of Sciences, King Khalid University, Abha, 61413 Saudi Arabia

Retraction of: *Scientific Reports* 10.1038/s41598-022-20239-y, published online 30 September 2022

The Editors have retracted this Article.

Following publication, the inclusion of several questioned references in the Article was brought to the attention of the Editors. Post-publication peer-review of the Article has uncovered further concerns such as the rationale of the work not being adequately justified, lack of clarity in the mathematical arguments and inferences, and inadequate validation and benchmarking. The Editors therefore no longer have confidence in the conclusions presented.

Tahir Mahmood disagrees with the retraction. Zeeshan Ali and Muhammad Aslam did not respond to the correspondence from Editors about this retraction.

